# Supplementary material for: A Global Screen for Assembly State Changes of the Mitotic Proteome by SEC-SWATH-MS
Source: Cell Syst. 2020 Feb 26;10(2):133–155.e6. doi: 10.1016/j.cels.2020.01.001 (PMC7042714; doi:10.1016/j.cels.2020.01.001)

Q9NWM8 | FKB14_HUMAN | FKBP14 FKBP22 UNQ322/PRO381
Monomer MW [kDa]: 24.172 Monomer expected elution fraction: 53

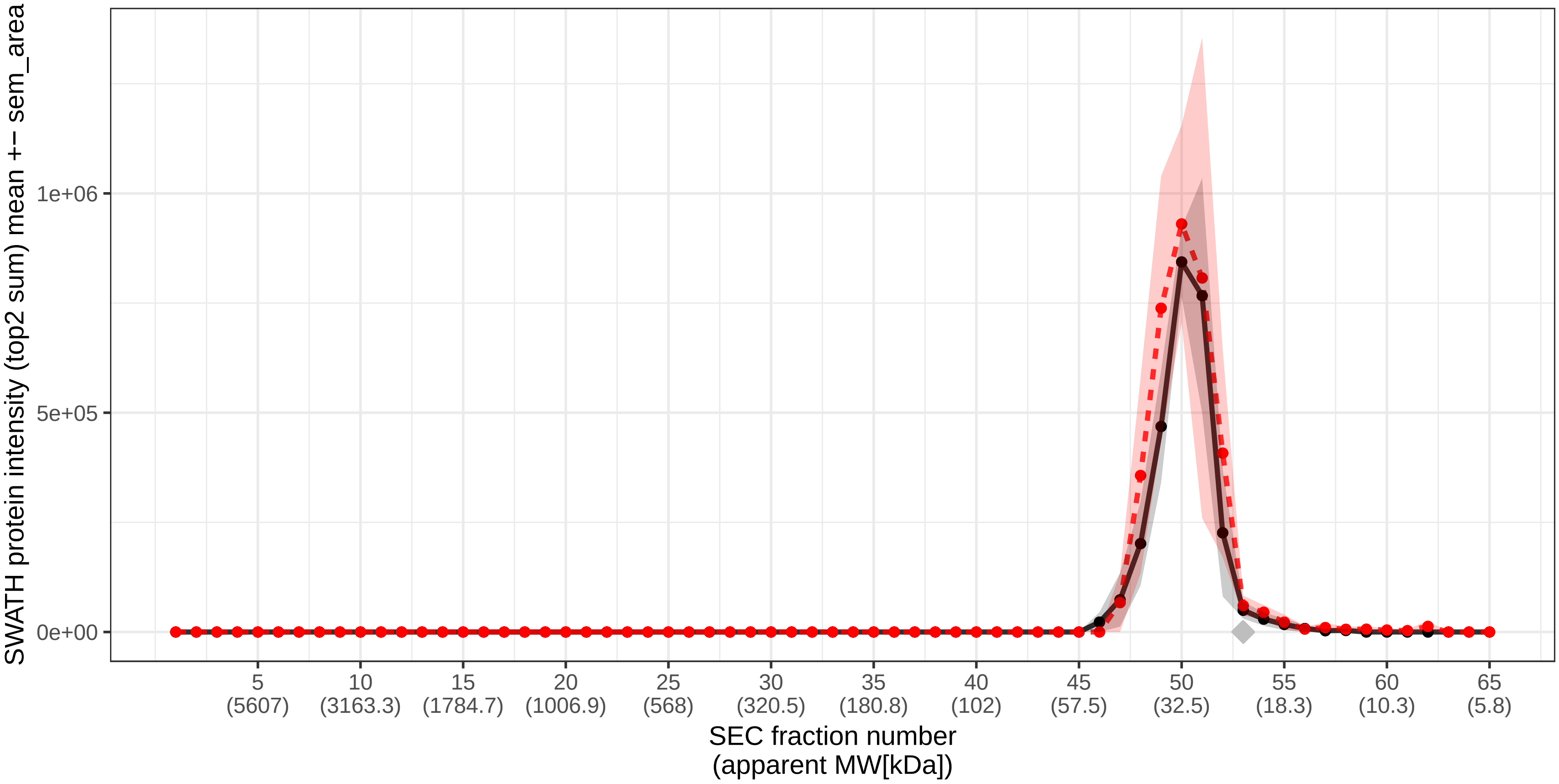

Supplement: Data S1. SEC-SWATH-MS Protein Chromatograms, Related to Figure 1 [file mmc6.zip › SECchrom_Q9NWM8_FKB14_HUMAN_FKBP14_FKBP22_UNQ322_PRO381.pdf]
